# Supplementary material for: Foxtrot migration and dynamic over-wintering range of an Arctic raptor
Source: eLife. 2024 Nov 8;12:RP87668. doi: 10.7554/eLife.87668 (PMC11548880; doi:10.7554/eLife.87668)
Supplement: Supplementary file 1. — (a) Table S1. The relationship between latitude/longitude and the day of the year during quick and slow phases of migration. The likelihood ratio test compares two candidate models: with (‘~doy’) and without the day of the year (‘~1’) as a fixed factor. (b) Table S2. The relationship between latitude/longitude and the day of the year during quick and slow phases of migration. Linear mixed-effect model, fixed effects. The response variable – latitude/longitude. Fixed effect – the day of the year (‘doy’). Random effects – individuals and year. (c) Table S3. The relationship between latitude/longitude and the day of the year during quick and slow phases of migration. Linear mixed-effect model, random effects. The response variable – latitude/longitude. Fixed effect – the day of the year. Random effects – individuals (‘bird’) and year (‘year’). (d) Table S4. The difference between the distance of slow and quick migrations. Linear mixed-effect model, post hoc results. The response variable – distance (km). Fixed effect – the type of migrations. Results of the post hoc comparison. (e) Table S5. The difference between the duration of slow and quick migrations. Linear mixed-effect model, post hoc results. The response variable – duration (days). Fixed effect – the type of migrations. Results of the post hoc comparison. (f) Table S6. The difference between the speed of slow and quick migrations. Linear mixed-effect model, post hoc results. The response variable – speed (km/day). Fixed effect – the type of migrations. Results of the post hoc comparison. (g) Table S7. The difference between the direction of the spring and the second phase of the winter migration and between the autumn and the first phase of the winter migration (two models). Linear mixed-effect models, post hoc results. The response variable in both models – direction (deg). Fixed effect – the type of migrations. Results of the post hoc comparison. (h) Table S8. The relationship between migration distance and [file elife-87668-supp1.docx]

# SUPPLEMENTARY MATERIAL

# Supplementary File 1

Foxtrot migration and dynamic over-wintering range of an arctic raptor

Ivan Pokrovsky^1^ *, Teja Curk^2^, Andreas Dietz^3^, Ivan Fufachev^4^, Olga Kulikova^5^, Sebastian Rößler^3^, Martin Wikelski^1,6^

^1^ Department of Migration, Max Planck Institute of Animal Behavior, Radolfzell, Germany

^2^ Leibniz Institute for Zoo and Wildlife Research, Berlin, Germany

^3^ Deutsches Zentrum für Luft- und Raumfahrt e.V. (DLR), Wessling, Germany

^4^ Institute of Biological Problems of the North, Magadan, Russia

^5^ Institute of Plant and Animal Ecology, Yekaterinburg, Russia

*^6^ Centre for the Advanced Study of Collective Behaviour, University of Konstanz, 78457 Konstanz, Germany*

* Corresponding author

E-mail: [ipokrovsky@ab.mpg.de](mailto:ipokrovsky@ab.mpg.de)

**a) Table S1**. The relationship between latitude/longitude and the day of the year during quick and slow phases of migration. The likelihood ratio test compares two candidate models: with (“~doy”) and without the day of the year (“~1”) as a fixed factor. For the fixed effects, see Table S2. For the random effects, see Table S3.

| **Migration** | **Model** | **df** | **AIC** | **BIC** | **logLik** | **dev** | **Chisq** | **p-value** |
| --- | --- | --- | --- | --- | --- | --- | --- | --- |
| Quick: spring  (latitude) | ~ 1 | 4 | 4290 | 4309 | -2141 | 4282 |  |  |
|  | ~ doy | 5 | 3612 | 3635 | -1801 | 3602 | 680 | <0.001 |
| Quick: fall  (latitude) | ~ 1 | 4 | 4816 | 4834 | -2404 | 4808 |  |  |
|  | ~ doy | 5 | 4277 | 4300 | -2133 | 4267 | 541 | <0.001 |
| Slow: 1^st^ phase  (latitude) | ~ 1 | 4 | 14916 | 14940 | -7454 | 14908 |  |  |
|  | ~ doy | 5 | 13690 | 13721 | -6840 | 13680 | 1228 | <0.001 |
| Slow: 1^st^ phase  (longitude) | ~ 1 | 4 | 19746 | 19771 | -9869 | 19738 |  |  |
|  | ~ doy | 5 | 18929 | 18960 | -9459 | 18919 | 820 | <0.001 |
| Slow: 2^nd^ phase  (latitude) | ~ 1 | 4 | 13561 | 13585 | -6777 | 13553 |  |  |
|  | ~ doy | 5 | 11726 | 11756 | -5858 | 11716 | 1837 | <0.001 |
| Slow: 2^nd^ phase  (longitude) | ~ 1 | 4 | 19250 | 19275 | -9621 | 19242 |  |  |
|  | ~ doy | 5 | 17482 | 17512 | -8736 | 17472 | 1770 | <0.001 |

**b) Table S2.** The relationship between latitude/longitude and the day of the year during quick and slow phases of migration. Linear mixed-effect model, fixed effects. The response variable – latitude/longitude. Fixed effect – the day of the year (‘doy’). Random effects – individuals and year. For the random effects, see Table S3. For the likelihood ratio test results to compare candidate models, see Table S1.

| **Migration** |  | **Estimate** | **Std.Error** | **t value** | **p-value** |
| --- | --- | --- | --- | --- | --- |
| Quick: spring  (latitude) | Intercept | 2.945 | 1.928 | 1.53 |  |
|  | doy | 0.477 | 0.014 | 34.56 | <0.001 |
| Quick: fall  (latitude) | Intercept | 201.875 | 4.872 | 41.44 |  |
|  | doy | -0.503 | 0.016 | -30.47 | <0.001 |
| Slow: 1^st^ phase  (latitude) | Intercept | 55.500 | 0.678 | 81.90 |  |
|  | doy | -0.026 | 0.001 | -38.55 | <0.001 |
| Slow: 2^nd^ phase  (latitude) | Intercept | 42.785 | 0.482 | 88.73 |  |
|  | doy | 0.046 | 0.001 | 50.03 | <0.001 |
| Slow: 1^st^ phase  (longitude) | Intercept | 46.186 | 1.764 | 26.18 |  |
|  | doy | -0.045 | 0.001 | -30.42 | <0.001 |
| Slow: 2^nd^ phase  (longitude) | Intercept | 18.566 | 1.518 | 12.23 |  |
|  | doy | 0.113 | 0.002 | 48.84 | <0.001 |

**c) Table S3.** The relationship between latitude/longitude and the day of the year during quick and slow phases of migration. Linear mixed-effect model, random effects. The response variable – latitude/longitude. Fixed effect – the day of the year. Random effects – individuals (‘bird’) and year (‘year’). For the fixed effects, see Table S2. For the likelihood ratio test results to compare candidate models, see Table S1.

| **Migration** | **Group** | **Variance** | **Std. Dev.** |
| --- | --- | --- | --- |
| Quick: spring  (latitude) | bird | 7.998 | 2.828 |
|  | year | 1.579 | 1.256 |
|  | residual | 7.054 | 2.656 |
| Quick: fall  (latitude) | bird | 25.858 | 5.085 |
|  | year | 12.766 | 3.573 |
|  | residual | 8.849 | 2.975 |
| Slow: 1^st^ phase  (latitude) | bird | 6.812 | 2.610 |
|  | year | 1.377 | 1.173 |
|  | residual | 2.968 | 1.723 |
| Slow: 1^st^ phase  (longitude) | bird | 34.222 | 5.850 |
|  | year | 14.999 | 3.873 |
|  | residual | 13.547 | 3.681 |
| Slow: 2^nd^ phase  (latitude) | bird | 1.921 | 1.386 |
|  | year | 0.959 | 0.979 |
|  | residual | 2.424 | 1.557 |
| Slow: 2^nd^ phase  (longitude) | bird | 19.047 | 4.364 |
|  | year | 10.699 | 3.271 |
|  | residual | 15.344 | 3.917 |

**d) Table S4.** The difference between the distance of slow and quick migrations. Linear mixed-effect model, post-hoc results. The response variable – distance (km). Fixed effect – the type of migrations. Results of the post hoc comparison. For the plot see Figure 1c.

| **Migration** | **difference** | **SE** | **df** | **t ratio** | **p-value** |
| --- | --- | --- | --- | --- | --- |
| Quick (spring) – Quick (fall) | 210 | 81 | 171 | 2,58 | 0,053 |
| Quick (spring) – Slow (first) | 528 | 87 | 164 | 6,04 | <0.001 |
| Quick (spring) – Slow (second) | 502 | 87 | 164 | 5,74 | <0.001 |
| Quick (fall) – Slow (first) | 318 | 80 | 164 | 4,00 | <0.001 |
| Quick (fall) – Slow (second) | 292 | 80 | 164 | 3,68 | 0,002 |
| Slow (first) – Slow (second) | -26 | 85 | 154 | -0,30 | 0,990 |

**e) Table S5.** The difference between the duration of slow and quick migrations. Linear mixed-effect model, posthoc results. The response variable – duration (days). Fixed effect – the type of migrations. Results of the post hoc comparison. For the plot, see Figure 1c.

| **Migration** | **difference** | **SE** | **df** | **t ratio** | **p-value** |
| --- | --- | --- | --- | --- | --- |
| Quick (spring) – Quick (fall) | 4 | 6 | 160 | 0,67 | 0,910 |
| Quick (spring) – Slow (first) | -103 | 6 | 153 | -16,99 | <0.001 |
| Quick (spring) – Slow (second) | -49 | 7 | 151 | -6,90 | <0.001 |
| Quick (fall) – Slow (first) | -107 | 6 | 154 | -19,36 | <0.001 |
| Quick (fall) – Slow (second) | -52 | 7 | 159 | -7,90 | <0.001 |
| Slow (first) – Slow (second) | 54 | 7 | 150 | 7,75 | <0.001 |

**f) Table S6.** The difference between the speed of slow and quick migrations. Linear mixed-effect model, posthoc results. The response variable – speed (km/day). Fixed effect – the type of migrations. Results of the post hoc comparison. For the plot, see Figure 1c.

| **Migration** | **difference** | **SE** | **df** | **t ratio** | **p-value** |
| --- | --- | --- | --- | --- | --- |
| Quick (spring) – Quick (fall) | -7 | 7 | 149 | -1.02 | 0.737 |
| Quick (spring) – Slow (first) | 92 | 7 | 143 | 12.05 | <0.001 |
| Quick (spring) – Slow (second) | 83 | 9 | 138 | 9.50 | <0.001 |
| Quick (fall) – Slow (first) | 99 | 7 | 143 | 14.33 | <0.001 |
| Quick (fall) – Slow (second) | 90 | 8 | 141 | 10.85 | <0.001 |
| Slow (first) – Slow (second) | -9 | 9 | 136 | -1.04 | 0.727 |

**g) Table S7.** The difference between the direction of the spring and the second phase of the winter migration and between the autumn and the first phase of the winter migration (two models). Linear mixed-effect models, posthoc results. The response variable in both models – direction (deg). Fixed effect – the type of migrations. Results of the post hoc comparison. For the plot, see Figure 1c.

| **Migration** | **difference** | **SE** | **df** | **t ratio** | **p-value** |
| --- | --- | --- | --- | --- | --- |
| Quick (spring) – Slow (second) | -50 | 3 | 70 | -16.08 | <0.001 |
| Quick (fall) – Slow (first) | -54 | 3 | 82 | -15,73 | <0.001 |

**h) Table S8.** The relationship between migration distance and the sex of the birds. The likelihood ratio test compares two candidate models: with (“~sex”) and without the sex (“~1”) as a fixed factor.

| **Model** | **df** | **AIC** | **BIC** | **logLik** | **dev** | **Chisq** | **p-value** |
| --- | --- | --- | --- | --- | --- | --- | --- |
| ~ 1 | 3 | 7673.8 | 7684.6 | -3833.9 | 7667.8 |  |  |
| ~ sex | 45 | 7674.7 | 7689.0 | -3833.3 | 7666.7 | 1.175 | 0.278 |

**i) Table S9.** The difference between vegetation land cover types crossed during quick (fall and spring) and slow (winter) migrations. General linear mixed-effect models. Results are given on the logit scale. For the plot, see Figure 2.

| **Type** | **Migration** | **Estimate** | **SE** | **z value** | **p-value** |
| --- | --- | --- | --- | --- | --- |
| Grassland | (Intercept) | -1.55 | 0.23 | -6.66 |  |
|  | Slow migration | 2.27 | 0.10 | 23.91 | <0.001 |
| Croplands | (Intercept) | -7.09 | 0.76 | -9.37 |  |
|  | Slow migration | 4.59 | 0.47 | 9.80 | <0.001 |
| Forests | (Intercept) | -0.22 | 0.15 | -1.49 |  |
|  | Slow migration | -3.04 | 0.11 | -28.49 | <0.001 |
| Urban | (Intercept) | -7.36 | 0.94 | -7.84 |  |
|  | Slow migration | 1.08 | 0.65 | 1.64 | 0.1 |

| **Month** | **Migration** | **Estimate** | **SE** | **z value** | **p-value** |
| --- | --- | --- | --- | --- | --- |
| October | Hyp SW – Hyp stay | -1.08 | 0.20 | -5.45 | <0.001 |
|  | Hyp SW – Real | -1.13 | 0.22 | -5.13 | <0.001 |
|  | Hyp stay – Real | -0.05 | 0.18 | -0.30 | 0.952 |
| November | Hyp SW – Hyp stay | -0.75 | 0.09 | -8.38 | <0.001 |
|  | Hyp SW – Real | -0.66 | 0.11 | -6.15 | <0.001 |
|  | Hyp stay – Real | 0.10 | 0.10 | 0.97 | 0.595 |
| December | Hyp SW – Hyp stay | -1.18 | 0.07 | -16.28 | <0.001 |
|  | Hyp SW – Real | -0.35 | 0.08 | -4.25 | <0.001 |
|  | Hyp stay – Real | 0.83 | 0.09 | 9.56 | <0.001 |
| January | Hyp SW – Hyp stay | -2.51 | 0.15 | -16.63 | <0.001 |
|  | Hyp SW – Real | 0.13 | 0.11 | 1.13 | 0.497 |
|  | Hyp stay – Real | 2.63 | 0.16 | 16.07 | <0.001 |
| February | Hyp SW – Hyp stay | -4.88 | 0.45 | -10.80 | <0.001 |
|  | Hyp SW – Real | -0.38 | 0.11 | -3.48 | 0.0015 |
|  | Hyp stay – Real | 4.50 | 0.46 | 9.86 | <0.001 |
| March | Hyp SW – Hyp stay | -4.87 | 0.14 | -34.14 | <0.001 |
|  | Hyp SW – Real | -1.81 | 0.11 | -16.94 | <0.001 |
|  | Hyp stay – Real | 3.06 | 0.14 | 22.43 | <0.001 |
| April | Hyp SW – Hyp stay | -2.45 | 0.14 | -17.98 | <0.001 |
|  | Hyp SW – Real | -2.47 | 0.15 | -1706 | <0.001 |
|  | Hyp stay – Real | -0.03 | 0.09 | -0.29 | 0.955 |

**j) Table S10.** The difference between snow cover conditions in the real situation (‘Real’) and two and two hypothetical situations – if birds spend winter in the place where they have arrived after fall migration (‘Hyp stay’) and if birds fly directly to the Southwest and stay there all winter (‘Hyp SW’). General linear mixed-effect models. Results are given on the logit scale. Results of the post hoc comparison. For the plot, see Figure 3b.
